# Characteristics and influencing factors of corneal higher-order aberrations in patients with cataract

**DOI:** 10.1186/s12886-023-03067-0

**Published:** 2023-07-12

**Authors:** Suowang Zhou, Xu Chen, Julio Ortega-Usobiaga, Hansong Zheng, Wenjing Luo, Biyue Tu, Yong Wang

**Affiliations:** 1 Aier Eye Hospital of Wuhan University (Wuhan Aier Eye Hospital), Wuhan, Hubei Province China; 2 New Bund Medical and Surgical Center, Sino United Health Clinics, Shanghai, China; 3 Shanghai Aier Eye Hospital, Shanghai, China; 4 Department of Cataract and Refractive Surgery, Clinica Baviera-AIER Eye Hospital Group, Bilbao, Spain

**Keywords:** Higher-order aberration, Cataract, Ocular biometric parameters, Spherical aberration

## Abstract

**Purpose:**

To observe the distribution characteristics of corneal higher-order aberrations (HOAs) in cataract patients, and analyze the relationship of HOAs with patients’ age and ocular biometric parameters.

**Methods:**

This retrospective study reviews the patients with cataract in Wuhan Aier Eye Department from January to August 2022. Root mean square (RMS) of the total HOA (tHOA), spherical aberration (SA), coma and trefoil aberration of the anterior cornea at central 4 and 6 mm optic zone were measured by the Wavefront Aberrometer (OPD-Scan III; Nidek Inc, Tokyo, Japan). The biometric parameters including axial length (AL), keratometry (K), central corneal thickness (CCT) and lens thickness (LT) were measured by swept-source coherence laser interferometry (OA-2000; TOMEY Corp, Aichi, Japan). Subgroup analyses and multiple linear regression analyses were used to determine whether HOAs were associated with age and ocular biometric parameters.

**Results:**

A total of 976 patients (976 eyes) were included, averagely aged 65 years. At central 4 and 6 mm optic zone, the mean RMS of tHOA were respectively 0.20 and 0.65 μm, the SA were 0.06 and 0.30 μm, the coma aberration were 0.11 and 0.35 μm, and the trefoil aberration were 0.12 and 0.30 μm. The tHOA decreased with age until 60 years and then started to increase afterwards. The tHOA, coma and trefoil aberration increased with corneal astigmatism. The tHOA, SA, and coma aberration differ among different AL groups, and emmetropes had the smallest tHOA, SA, and coma aberration.

**Conclusions:**

With increasing age, the value of tHOA decrease first and started increasing at 60 years. The trends of corneal HOAs are consistent with corneal low-order aberrations. The values of tHOA, SA and coma aberration were the smallest in emmetropic eyes.

**Supplementary Information:**

The online version contains supplementary material available at 10.1186/s12886-023-03067-0.

## Introduction

Human vision quality is closely related to ocular aberrations. As the human eye is not in an ideal sphere, the optical deviation along a certain ray from the perfect spherical wavefront is defined as wavefront aberration. Zernike polynomial is commonly used to quantitatively evaluate wavefront aberration of the human eye [1]. Among them, wavefront aberrations above the third order are defined as higher-order aberrations (HOAs), which are not correctable with sphere and cylinder corrections. HOAs may deteriorate visual acuity by resulting in decreased contrast sensitivity, glare, starburst and halos [2–4].

As we know, ocular HOAs are mainly derived from corneal and crystalline lens. And for young individuals, an aberration from the corneal and crystalline lens formed a mutual compensation relationship, [5] while ageing could disrupt this balance, [6] which could account for the optical deterioration in cataract patients. A better understanding of the causes of optical deterioration within the cataract patients will improve the maintenance of vision quality in the elderly.

In recent years, the application of wavefront-sensing devices made the measurement of HOAs possible and HOAs have been the topic of many recent studies. However, the major HOA determinants are still not fully understood. We conducted this study to investigate age-related changes in corneal HOAs and other ocular biometric parameters that may influence HOAs. It is the first study that investigate the distribution and influencing factors of corneal HOAs in cataract patients with a big sample size.

## Methods

In this retrospective study, we included the patients who are going to undergo cataract surgery in Wuhan Aier Eye Hospital (Hubei, province of China) from January to August 2022. Exclusion criteria included a history of ocular surgery, corneal scattering, or other corneal pathology. Subjects with missing or insufficient data were also excluded. If the both eyes of the patients were eligible, data from only the right eye were used to avoid the use of interdependent data between 2 eyes from the same subject.

Preoperative measurements were collected. The biometric parameters including axial length (AL), mean keratometry (MK), central corneal thickness (CCT), and anterior chamber depth (ACD) were measured by swept-source coherence laser interferometry (OA-2000; TOMEY Corp, Aichi, Japan). Corneal HOAs were obtained using the Wavefront Aberrometer (OPD-Scan III; Nidek Inc, Tokyo, Japan). The scanner is a multifunction instrument that integrates Placido-based corneal topography with wavefront aberrometry of the entire eye. The time difference of the reflected light to stimulate an array of photodetectors is translated to a refractive wavefront map. Measurements were taken by a single experienced technician, and all patients were examined twice if a consistent result had been obtained. If not, the patients were examined more than twice until accurate readings were obtained.

Anterior corneal higher order aberrations were measured at the central 4.0 and 6.0 mm optic zone. Based on Zernike coefficients, we calculated each root mean square (RMS) of total HOA (tHOA, square root of the sum of the squared coefficients from Z6 to Z44), spherical aberration (SA, square root of the sum of the squared coefficients of Z40), coma (square root of the sum of the squared coefficients of Z3 ± 1), trefoil (square root of the sum of the squared coefficients of Z3 ± 3).

To compare the distributions of corneal aberrations among different age, we divided the patients into 5 subgroups: 40–49, 50–59, 60–69, 70–79 and ≥ 80 years old. Likewise, to evaluate the relationship between corneal aberrations and corneal astigmatism, the patients were divided into 3 subgroups according to corneal astigmatism (calculated by steep keratometry and flat keratometry measured through OA-2000): <1D, 1D-1.99D and ≥ 2D [7]. In addition, to estimate the relationship between corneal aberrations and AL, patients were divided into 3 groups according AL: hyperopic group: AL < 22 mm; emmetropic group: 22 mm ≤ AL ≤ 25 mm;myopic group: AL > 25mm [8].

The statistical analysis was performed using SPSS 22.0 (IBM, Chicago, IL, USA). All parameters were proved non-normally distributed through the Kolmogorov-Smirnov test of normality. Thus, linear regression and Spearman correlation coefficients were used to compare groups and values. A multilinear regression model, consisting of ocular parameters associated with anterior corneal HOAs at the central 4 mm optic zone and SA at the central 6-mm optic zone, was tested. These parameters included age, AL, MK, CCT, and ACD. A *p*-value < 0.05 was considered statistically significant.

## Results

A total of 976 patients (976 eyes) were included (382 males and 594 females), aged 65 (range, 55–72) years. Mean AL was 24.73 ± 2.77 mm, mean CCT was 522 ± 34 μm, MK was 43.74 ± 1.93 diopter (D), mean ACD was 3.21 ± 0.44 mm (Table 1).

**Table 1:** Demographic characteristics and basic ocular parameters. SD: standard deviation; y: years; AL: axial length; CCT: corneal central thickness; MK: mean keratometry, D: diopters; ACD: anterior chamber distance

	Mean	SD
Age, y	63.52	10.94
Sex, male/female	382/594	
AL, mm	24,73	2.77
CCT, µm	522.06	34.81
MK, D	43.74	1.93
ACD, mm	3.21	0.43

**Fig. 1:**
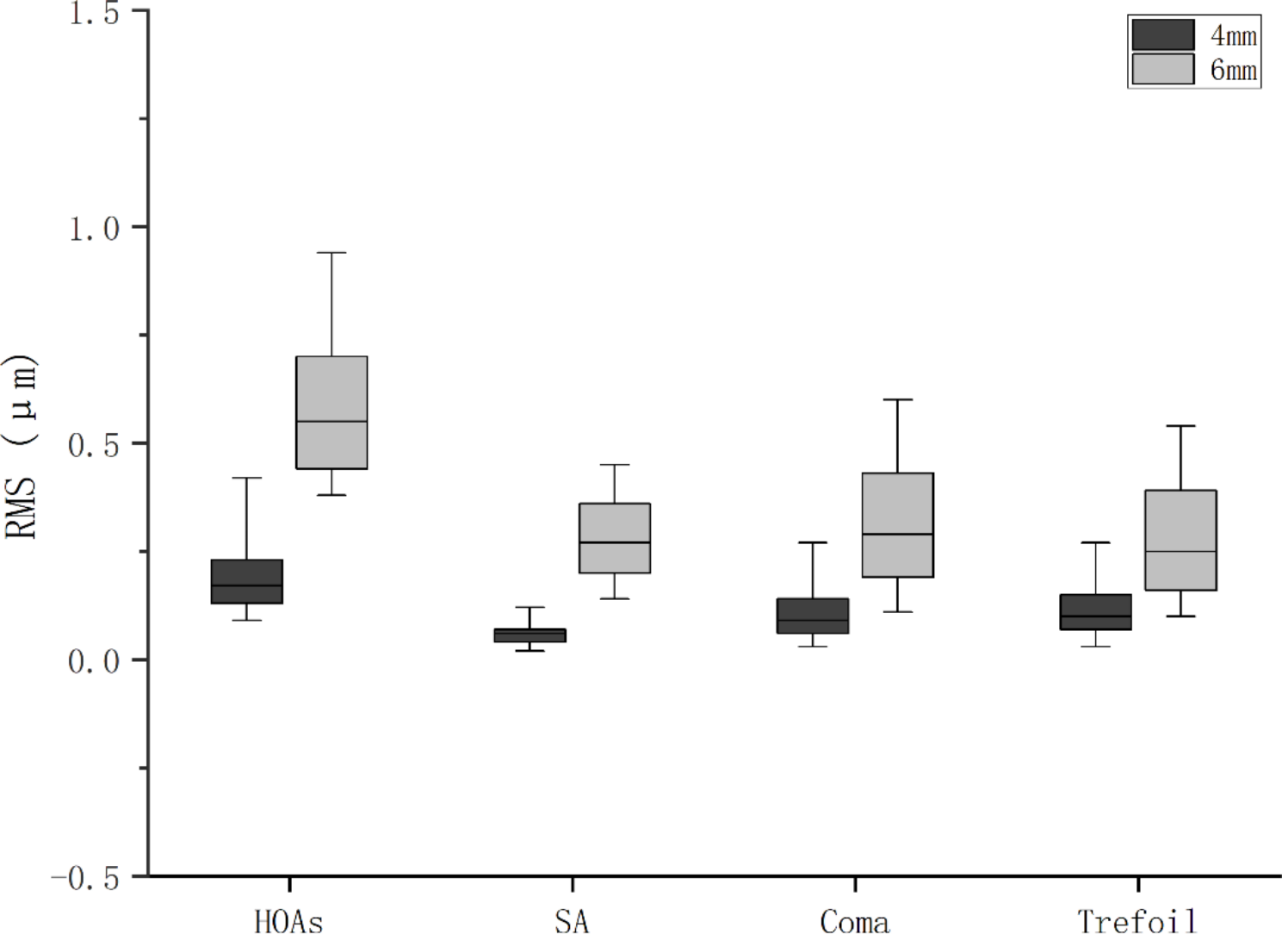
Comparisons of different higher-order aberrations (HOAs) between 4 and 6 mm corneal zone. SA = spherical aberration

At central 4 and 6 mm optic zone, the mean RMS values of tHOA were respectively 0.20 and 0.65 μm, SA was respectively 0.06 and 0.30 μm, coma aberration were respectively 0.11 and 0.35 μm, trefoil aberration was respectively 0.12 and 0.30 μm. RMS of corneal HOAs between central 4 and 6 mm optic zone were significantly different (P < 0.001) (Table 2; Fig. 1).

**Table 2:** Root Mean Square (RMS) values of corneal higher-order aberrations (HOAs). SA: spherical aberration

Aberration	4 mm zone	6 mm zone	*P*
tHOA	0.20 ± 0.12	0.65 ± 0.38	< 0.01
SA	0.06 ± 0.04	0.30 ± 0.18	< 0.01
Coma	0.11 ± 0.09	0.35 ± 0.27	< 0.01
Trefoil	0.12 ± 0.12	0.30 ± 0.23	< 0.01

**Fig. 2:**
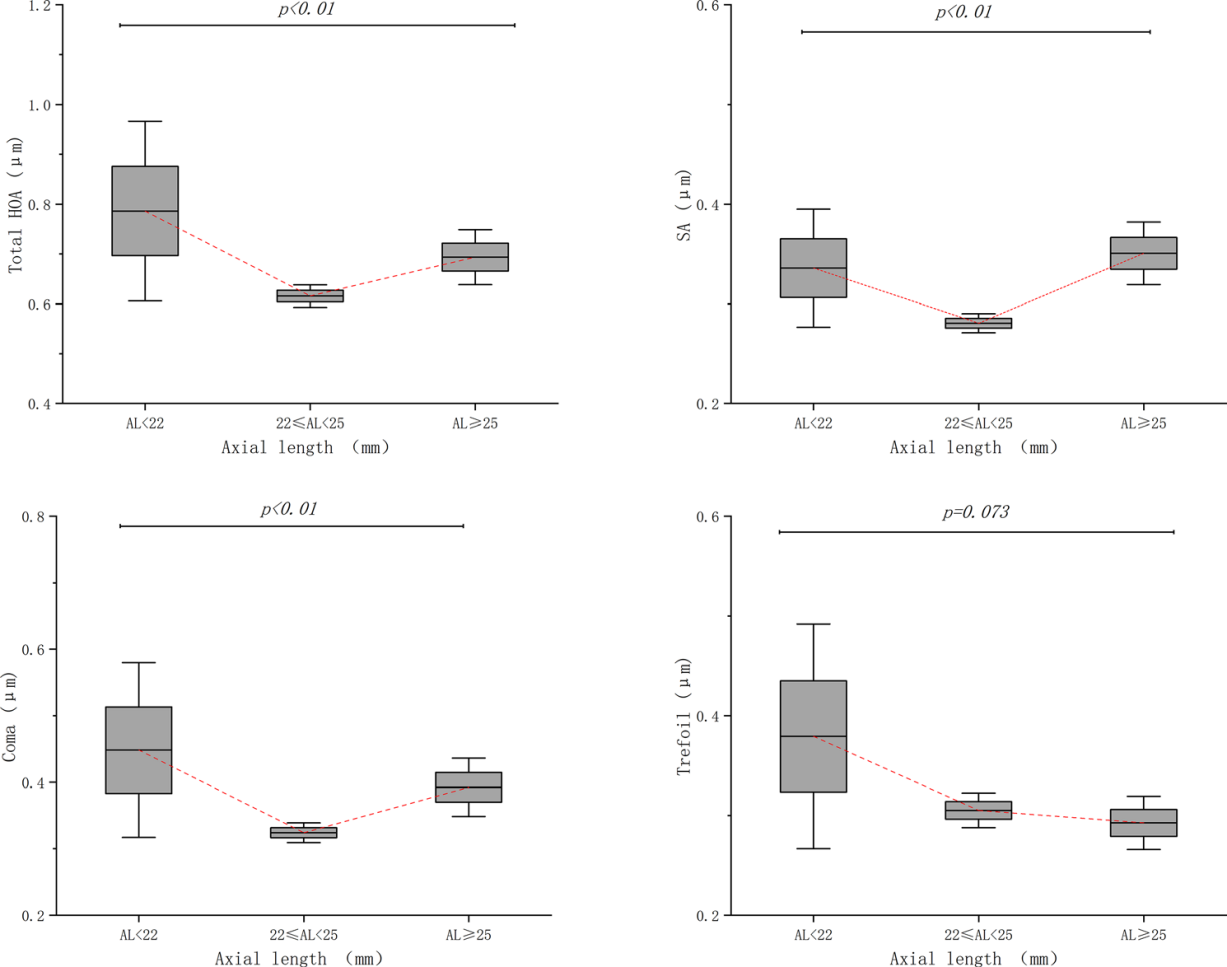
Distribution of corneal higher-order aberrations (HOAs) at central 6 mm optic zone stratified by axial length (AL).

At the central 6 mm optic zone, the RMS value of tHOA, SA, and coma aberration changed significantly among AL subgroups (*p < 0.01*). Emmetropic eyes have the lowest mean values and minimum range of variation of tHOA, SA, coma and trefoil aberration, compared with the other two groups (see Fig. 2).

**Fig. 3:**
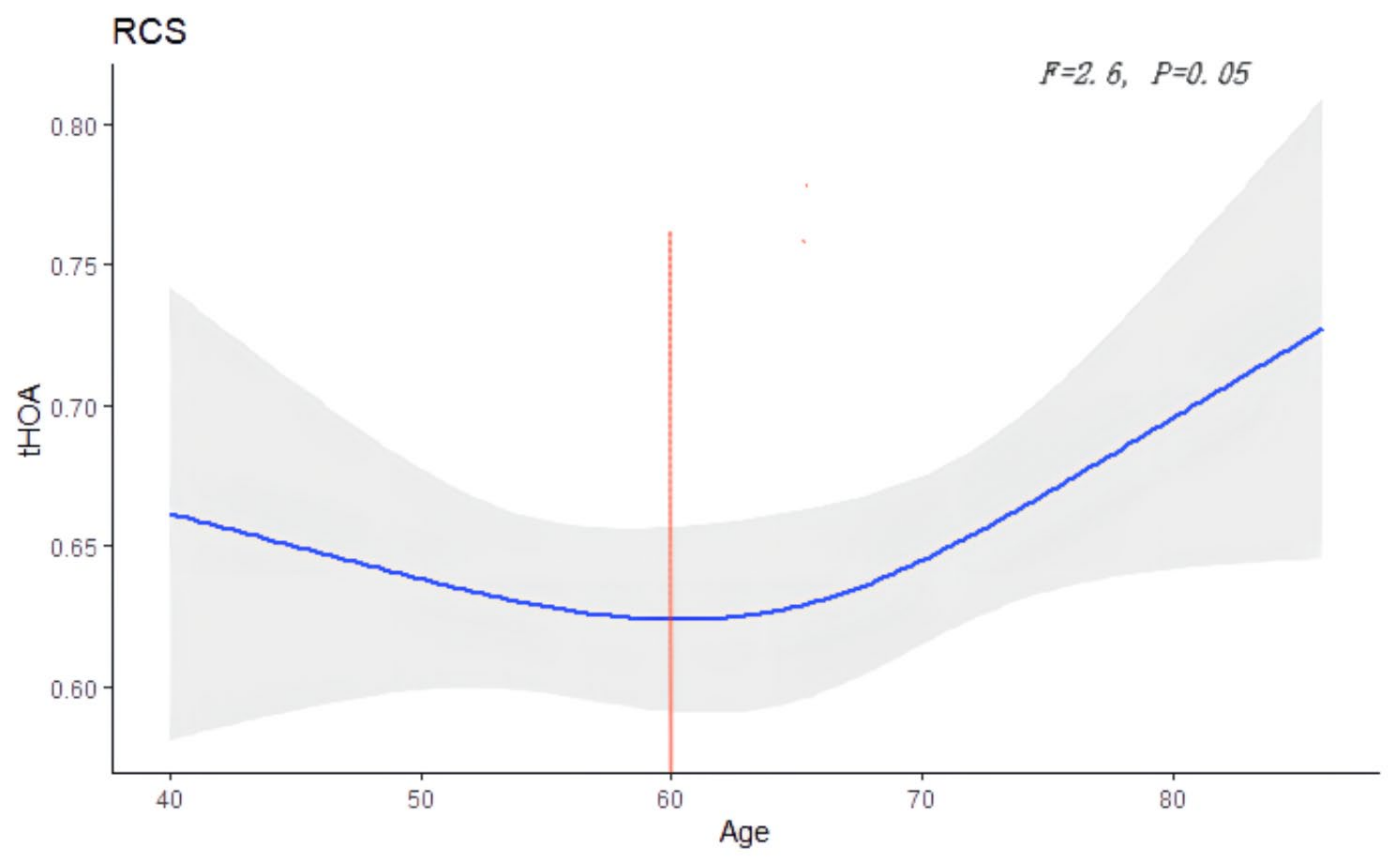
Association of total higher-order aberrations (tHOA) with age. Mean values are indicated by solid lines and 95%; CIs by shade areas

Supplemental Fig. 1 displayed the distribution of tHOA, SA, coma and trefoil RMS at the central 6 mm optic zone, stratified by age. The RMS of tHOA, coma and trefoil aberration among different age group were significant different (all *p < 0.01*) and the maximum was found in the group older than 80 years, while no significant difference was found in SA (*p = 0.172*). We also used restricted cubic splines to flexibly model and visualize the relation between tHOA and age. The tHOA decreased with age until 60 years and then started to increase afterwards (p for non-linearity = 0.05) (Fig. 3).

**Fig. 4:**
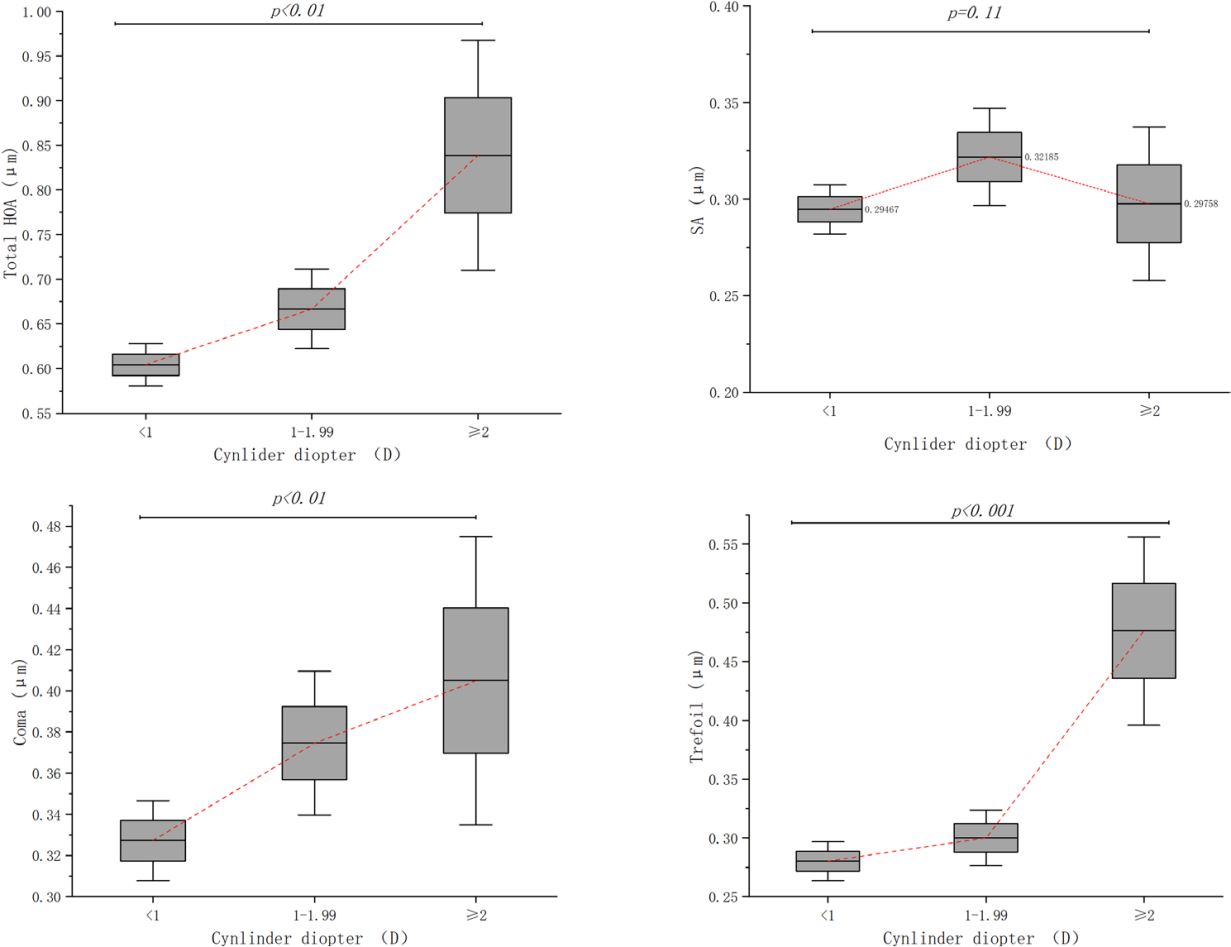
Distribution of corneal higher-order aberrations (HOAs) at central 6 mm optic zone stratified by corneal astigmatism

Among different astigmatism subgroups, significant differences were found in RMS values of tHOA, coma, and trefoil aberration (*p < 0.01*). (see Fig. 4) THOA, coma and trefoil aberration increased with corneal astigmatism.

Associations between corneal HOAs and age, AL, Km, CCT and ACD were analyzed by multiple linear regression analysis, which is summarized in Table 3. The tHOA was positively affected by age, and negatively affected by MK and CCT; SA was only negatively affected by Km and CCT; Coma aberration was positively affected by age and negatively affected by MK and CCT; Trefoil aberration was only positively affected by age. The multiple regression formulas are as follows:

- tHOA (6 mm) = 3.038 + 0.103Age – 0.217MK – 0.175CCT (R2 = 0.091; p < 0.01).
- SA (6 mm) = 2.135–0.301 Km – 0.245CCT (R2 = 0.172; p < 0.01).
- Coma (6 mm) = 1.966 + 0.077Age + 0.112AL – 0.191MK – 0.179CCT (R2 = 0.088; p < 0.01).
- Trefoil (6 mm) = 0.102 + 0.166 Age (R2 = 0.025; p = 0.001).

**Table 3:** Multiple linear regression analysis between high order aberrations (HOAs) at central 6 mm optic zone and age, axial length (AL), mean keratometry (MK), corneal central thickness (CCT) and anterior chamber distance (ACD). SA: spherical aberration

Dependent variable	Independent variable	Standard coefficient		P value
tHOA R^2^ = 0.091 p < 0.01	Age AL		0.103 0.069	0.003 0.067
	MK		-0.217	< 0.001
	CCT ACD		-0.175 -0.021	< 0.001 0.555
SA R^2^ = 0.172, p < 0.01	Age AL MK		-0.003 0.030 -0.301	0.925 0.405 < 0.001
	CCT ACD		-0.245 0.047	< 0.001 0.168
Coma	Age		0.077	0.027
R^2^ = 0.088; p < 0.01	AL		0.112	0.003
	MK		-0.191	< 0.001
	CCT ACD		-0.179 -0.041	< 0.001 0.246
Trefoil R^2^ = 0.025; p < 0.01	Age AL MK		0.166 -0.003 -0.009	< 0.001 0.929 0.784
	CCT ACD		-0.003 0.025	0.932 0.507

## Discussion

With continuous improvements in ocular biometric measurements and innovation of cataract surgery technology, a good outcome of a cataract surgery not merely implies good visual acuity, but also good vision quality. Some studies reported postoperative visual complaints can be attributed to an elevated level of HOAs, especially, SA and coma [9,10]. The increased SA has been associated with glare and halos, coma has been correlated with double vision, and trefoil has been associated with starburst [11]. In general, all Zernike coefficients are larger for corneal aberrations than for total wavefront aberrations, suggesting a compensatory effect from intraocular structures. For cataract patients, the lens is going to be replaced by an IOL. Thus, a new aberration balance between the cornea and IOL plays a vital role in vision quality. Hence, comprehensive knowledge of the distribution of corneal HOAs is meaningful for cataract surgery design and helpful for postoperative vision quality estimation. This work aimed to provide a comprehensive analysis of several types of corneal HOAs and their relationships with age and other ocular biometric parameters in senior Chinese patients with cataracts.

### Distribution of tHOA and SA

The mean RMS value of tHOAs in this study, measured at an optical zone of 6 mm, was 0.65 μm, consistent with the values reported in other studies of Chinese patients [12,13]. At the central 4 mm corneal zone, the mean RMS value of tHOA was 0.20 μm, significantly lower than at central 6 mm. And among them, 88.63% of patients had tHOA < 0.3 μm, and 97% <0.5 μm. Previous studies show that increased HOAs may deteriorate the visual outcomes of multifocal intraocular lenses (MIOL) [14,15]. Therefore, we found that the majority of cataract patients are eligible for MIOL in HOAs, while there are still a fraction of people who are inappropriate for MIOL due to HOAs.

SA is a type of HOAs and is caused by a difference in focus between central rays and peripheral rays that reach the retina at the same time. In clinical practice, cataract surgeons may prefer aspheric IOLs to improve contrast sensitivity and visual quality [10, 16−19]. In our study, the mean RMS value of corneal SA was 0.30 μm at the central 6 mm optic zone at the mean age of 63.52 ± 10.94 years, which was close to the result reported by Lai et al. in a Taiwanese population (0.307 μm) [20]. Furthermore, our result was higher than that of the Japanese populations [21] (0.23 μm), but lower than that in the Italian [22] (0.328 μm) population. According to another study based on the Chinese population in Beijing, [23] the mean corneal SA was 0.413 ± 0.161 μm. Ruling out the race factor, we infer that the mean age of the Beijing [23] population (73 years) was older than our study (63 years). Besides, the different devices used to determine corneal aberrations may also account for this difference.

Previous studies have suggested that lowering postoperative SA can achieve an improvement in visual quality and contrast sensitivity [24,25]. The mean corneal SA for our study was 0.30 μm. Another study based on population in Shanghai show the primary SA was 0.36 μm in patients with AL longer than 26mm [7]. As it knows, the most commonly used aspheric IOL with SA of -0.27 μm can compensate for corneal SA and achieve moderate residual positive SA, which results in better contrast sensitivity and ideal visual quality.

### Relationship between corneal HOAs and age

We investigated the relationship between age and tHOA, coma aberration, trefoil aberration, and SA. We found statistical differences in coma aberration, trefoil aberration and tHOA among different age groups, whereas no difference in SA. Wei, S. et al. [13] reported similar findings, showing that coma and tHOA correlate with age, while SA does not. Fujikado et al. [26] reported that ocular tHOA, including ocular SA, increased significantly after 50 years, but there was no correlation between age and corneal SA. Lyall et al. [27] also found that ocular SA increase with age, while corneal SA does not, indicating that the increase of ocular SA mainly results from the increase of internal optical aberrations. These results suggest that tHOA, coma, and trefoil increase with age, possibly induced by age-related changes in the anterior surface of the cornea. Because coma aberration consists of tilt and asymmetry [28], these results imply that corneas become less symmetric with age. On the contrary, SA consists of a central-to-peripheral balance of the corneal curvature [29]. SA did not show any age-related changes, indicating that the central-to-peripheral balance of the corneal curvature is not significantly affected by age. In addition, the increase in tHOA with aging may be a factor that leads to a deterioration in their visual quality, which can help to explain why many early cataract patients often complain of obvious discomfort like photophobia, glare, poor night vision, although still maintained a good daily vision.

In order to further investigate the association between age and corneal HOAs, restricted cubic spline was used to flexibly model and visualize the non-linear relation of tHOA and age. And a ‘U’ shape was observed: tHOA decreased with age until 60 years and then started to increase afterwards. Interestingly, another study investigated the relationship between corneal astigmatism and age among the Chinese senile cataract population older than 50, and also found a ‘U-like’ shape between age and corneal astigmatism: the turning point was at 65 years old. This indicated both corneal low-order and higher-order aberrations change with age, not in a linear correlation, but in a ‘U’ shaped non-linear correlation.

To further investigate the potential correlation between low-order aberrations (like astigmatism) and HOAs, we made a subgroup analysis according to astigmatism. We observed that tHOA, coma and trefoil increase with astigmatism. In previous study, authors evaluated HOAs in subjects with different refractive errors and they found that total HOAs, coma and trefoil were highest in astigmatic eyes while spherical aberrations were lowest in the astigmatic patients [30]. In another study conducted by Chenge et al. [31] reveals that astigmatic eyes tend to have a larger tHOA than non-astigmatism eyes. SA is a kind of HOA that caused by a difference in focus between central rays and peripheral rays, which is centrally symmetrical. While astigmatism is caused by the different refractive power in different meridians and the coma and trefoil aberration belong to irregular astigmatism, it’s reasonable that the changing trend of coma and trefoil aberration are consistent with astigmatism. It’s suggested that patients with higher astigmatism tend to have a higher level of HOAs. Hence, a comprehensive corneal aberration examination was recommended for high astigmatism patients, which can help surgeons to select the appropriate IOL type and make a preoperative visual quality assessment.

### Relationship between corneal HOAs and ocular biometric parameters

In addition to age, the relationship between HOAs and other factors has also been explored. Philip et al. [32] studied the tHOA and corneal topography of myopic, emmetropic, and hyperopic eyes of 675 adolescents and found no difference for anterior corneal SA among different refractive statuses. However, Llorente et al. [33] found that the average corneal SA was significantly higher in the hyperopic group than in the myopic one.

Accommodation can influence the corneal curvature, and especially, change corneal HOAs [34,35]. Mohamed et al. [36] observed that neither CCT nor peripheral corneal thickness (PCT) was significantly associated with corneal aberrations. Besides, Arba Mosquera and de Ortueta [37] demonstrated that corneal SA correlate well with Q values.

In the current study, we found that SA, coma aberration and tHOA differs among different AL subgroups. The emmetropic group had the lowest RMS value of tHOA, SA and coma aberration, while hyperopic group had the highest value of tHOA and coma aberration, and there is no statistical difference in trefoil aberration among different AL subgroups. In this regarding, there are several contrasting results. Shimozono et al. [21] found a significantly negative correlation between AL and corneal SA (r=-0.135). Kirwan et al. [38] demonstrated that third-order aberrations were higher in myopia [38]. Llorente et al. [33] reported that third-order aberrations (coma and trefoil) and SA were higher in young hyperopic eyes than in myopic eyes, while intraocular SA was not significantly different between these two groups. For hyperopic eyes, the anterior segment is relatively narrow and thus leads to corneal deformation, which may be related to the increase of HOAs. Furthermore, total RMS coma constituted a larger proportion of HOAs in hyperopes. It is known that angle kappa is larger in hyperopes compared to myopes and emmetropes, and a larger displacement of the pupillary axis from the visual axis is responsible for higher levels of coma among hyperopes. Remarkably, although we did observe differences among different AL groups, AL was not stronger enough to predict corneal HOAs in a multiple regression model.

In the multiple linear regression model of corneal HOAs, MK and CCT play the most important role in predicting corneal HOAs, which are negatively correlated with tHOA, SA and coma. Nonetheless, the R square of HOAs prediction based on ocular biometric parameters is relatively low. We refer to several similar articles and found the R square is generally low [12,39]. We think the possible reason could be as follows. Firstly, the ocular parameters may not be the most sensitive predictors of HOA. Secondly, we think it is also related to the huge individual difference of HOA. Due to the various influencing factors and individual differences, it is hard to establish an ideal prediction model under the current conditions based on a small sample size. In addition, the lack of equal pupil size in the measured eyes, different age distributions, and various measuring devices in each study necessitates comparisons between studies be made with caution. Hence, more efforts need to be attributed to standardize the measurement method and a personalized measurement of wavefront aberration is required at present.

Limitations of this study include the fact that we studied only a limited sample size with all the subjects enrolled at the same study centre. In addition, although human parameter measurements typically exhibit a normal distribution, we obtained non-normal distribution for the ophthalmic parameter values assessed. A possible explanation might be the age of the patients, who were elderly (63 ± 10.94 years).

In summary, approximately 90% of total aberrations are caused by the cornea; [21] therefore, corneal wavefront analysis is an important tool for evaluating vision quality. The mean value of tHOA and SA at an optical zone of 6 mm was 0.65 μm and 0.30 μm, respectively. With increasing age, the value of tHOA decreased first and started increasing at 60 years. The tHOA, coma and trefoil aberration increased with astigmatism. Among different AL groups, emmetropes had the lowest RMS value of tHOA, SA and coma aberration, while hyperopes had the highest RMS value of tHOA and coma aberration.

## Electronic supplementary material

Below is the link to the electronic supplementary material.

**Supplemental Figure 1** Distribution of corneal higher-order aberrations (HOAs) at central 6mm optic zone stratified by age.

## Data Availability

The datasets analyzed during the current study are not publicly available but are available from the corresponding author at a reasonable request.
