# Supplementary material for: Characteristics and influencing factors of corneal higher-order aberrations in patients with cataract
Source: BMC Ophthalmol. 2023 Jul 12;23:313. doi: 10.1186/s12886-023-03067-0 (PMC10337181; doi:10.1186/s12886-023-03067-0)
Supplement: Supplementary file 1 — Supplemental Figure 1 Distribution of corneal higher-order aberrations (HOAs) at central 6mm optic zone stratified by age. [file 12886_2023_3067_MOESM1_ESM.docx]

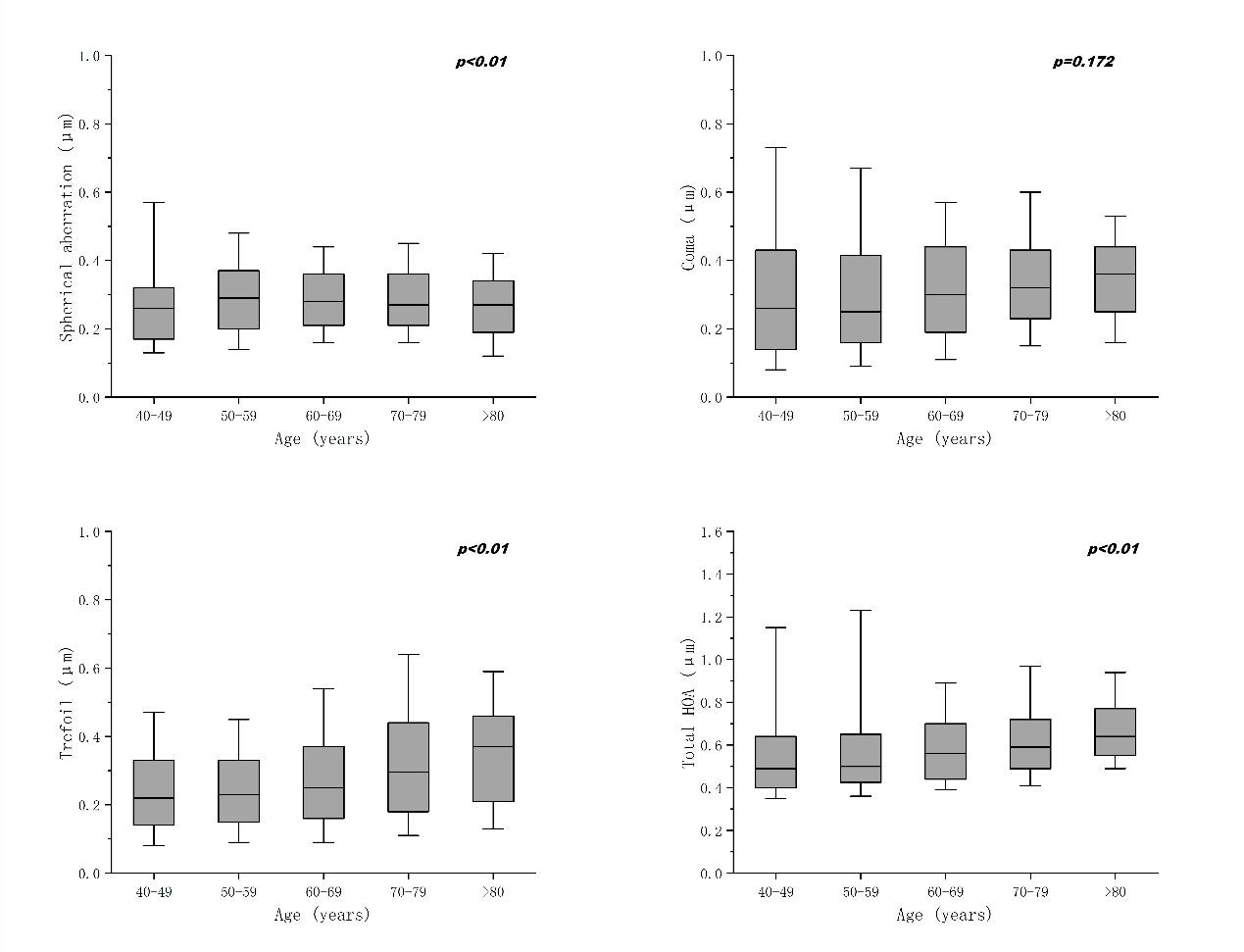


Supplemental Figure 1 Distribution of corneal higher-order aberrations (HOAs) at central 6mm optic zone stratified by age.
